# Perception of social experiences and cortical thickness change together throughout early adolescence: Findings from the ABCD cohort

**DOI:** 10.1162/IMAG.a.27

**Published:** 2025-06-06

**Authors:** Kathryn E. Bates, Ayla Pollmann, Rogier A. Kievit, Delia Fuhrmann

**Affiliations:** Institute of Psychiatry, Psychology, and Neuroscience, King’s College London, London, United Kingdom; Cognitive Neuroscience Department, Donders Institute for Brain, Cognition and Behaviour, Radboud University Medical Center, Nijmegen, The Netherlands

**Keywords:** adolescence, social experiences, cortical development, structural equation modelling, ABCD, puberty

## Abstract

Early adolescence is a dynamic period of social and brain development amid rapid hormonal and puberty changes. We examined how differences and changes in positive social experiences and cortical thickness co-develop from age 9–11 and 11–13 years in the ABCD cohort (N~12,000). We used bivariate latent change score models to capture cortical development (modeling mean whole-brain cortical thickness) and positive social experiences (modeling caregiver monitoring, family cohesion, prosocial behavior, number of friends, school engagement, school involvement, and neighborhood safety). We found evidence for correlated change, such that a greater reduction in positive social experiences was associated with a greater decrease in cortical thickness (est = 2.54**,**SE = .54, z = 4.74,*p*< .001, standardized effect size = .08), which did not differ between males and females in early and late puberty stages. We found mixed evidence for sex-specific relationships between puberty stage and social experiences, highlighting the need to better understand males’ puberty and social experiences in early adolescence. The evidence supports a transactional model of development in that positive social experiences and cortical thickness change together throughout early adolescence. The findings also highlight the importance of supporting youth in early adolescence through school transitions.

## 1 Introduction

Adolescence is a dynamic period of biological, social, and psychological development. It is defined as the period of development after childhood and before obtaining a stable social role, usually around 24 years of age (Sawyer et al., 2018). Puberty is a biological marker of the start of adolescence. It comprises a complex set of biological processes and neurological changes that result in the development of both primary and secondary sex characteristics, preparing individuals for reproduction (Blakemore et al., 2010). The time at which young people reach different stages of puberty varies between individuals and populations. For instance, in the United States, boys show signs of puberty at age 10 on average, and girls between age 9 and 10 years (Mendle et al., 2019).

After the onset of puberty, early adolescents face unique challenges. These include greater academic demands and ongoing psychosocial development, such as developing a sense of self (van der Cruijsen et al., 2023). During this phase of life, social experiences appear more salient, the social environment becomes broader, and many adolescents experience more complex peer and intimate relationships (Blakemore & Mills, 2014;Cheng et al., 2024). In early adolescence, an individual’s perception of their environment changes, and this is found to be related to later outcomes. Research thus far has focused on how perceptions of the school environment in early adolescence fluctuate around the transition to secondary/high school. Studies have found a decline in school engagement around the transition to high school and through to later adolescence (Wang & Dishion, 2012;Way et al., 2007). Adolescents’ school well-being—how content they are at school—in their first year of high school has been found to predict higher academic achievement later in the year through high-quality relationships with parents (Kiuru et al., 2020). Parenting practices, such as caregiver monitoring, during the transition to high school have, in turn, been found to predict later academic achievement and the likelihood of university/college enrolment (Hill & Wang, 2015).

This literature does not fully capture the multiple facets of early adolescents’ social environment and increasing independence from caregivers, where perceptions of peer relationships and the surrounding community are becoming more salient. Evidence shows friendships become more reciprocal and improve in quality throughout adolescence (Burnett Heyes et al., 2015). Friendship quality is, in turn, positively associated with well-being in adolescence (Alsarrani et al., 2022). Lower associations between stressful life events and mental health are found in early adolescence, where young people (12–13 years) report greater neighborhood safety (Kingsbury et al., 2020). This evidence demonstrates the importance of a broader conceptualization of adolescents’ social environment.

These extensive social changes during early adolescence occur around the same time as accelerated cortical development compared to middle childhood and late adolescence (Fuhrmann et al., 2022). Several neuroimaging studies have shown cortical thinning, associated with neurobiological processes such as synaptic pruning and increased myelination of deeper layers (Natu et al., 2019), extends into early adulthood. Longitudinal evidence shows that whole-brain cortical thickness increases up to early childhood (around 2 years old) and then declines through to late adulthood (Bethlehem et al., 2022). New research with twelve waves of longitudinal data between ages 7 and 21 years has suggested cortical thinning is nonlinear, with the fastest thinning seen around 14 years of age, and there are individual differences in the age of fastest thinning (Fuhrmann et al., 2022). Variability in trajectories was predicted by sex in that the age of fastest thinning was earlier in females compared to males. Evidence from animal models has shown the influx of gonadal hormones during puberty influences thinning of the prefrontal cortex, and synaptic reduction in female rodents is associated with pubertal timing (Delevich et al., 2021). Pubertal timing might, therefore, shape adolescent neurodevelopment. Research in humans following boys and girls from the pre-pubertal stage for 2 years found cortical thinning trajectories were predicted by individual differences in sex hormones during this period (Herting et al., 2015). A more recent study found evidence for non-linear trajectories of cortical thinning between ages 8.5 and 14.5 years, which varied depending on sex and pubertal tempo (Vijayakumar et al., 2021). A 2022 review investigating the role of puberty in structural brain development pointed out that we have a limited understanding of how structural brain development during puberty is related to real-life behaviors (Goddings et al., 2019). There is some evidence to suggest differences in susceptibility to the environment, such as social experiences, may depend on the timing of puberty onset (Laube & Fuhrmann, 2020). However, little is known about the relationship between puberty and cortical thinning in early adolescence in the context of social experiences, despite evidence that social experiences are a prominent developmental feature of early adolescence (Blakemore & Mills, 2014;Cheng et al., 2024).

The current literature on how social experiences interact with brain development has focused on early life experiences. Specifically, there is evidence to suggest that experiences during early life, including adverse experiences and cognitive enrichment, impact later brain structure and function (McLaughlin et al., 2019;Teicher et al., 2016;Tooley et al., 2021). This literature has influenced policy and investment in preventative measures for young children (Edwards et al., 2019). Alongside this research in the early years, we need to understand how adolescent social experiences shape cortical development. When associations between social experiences and cortical development during adolescence have been investigated, studies have mostly focused on adverse experiences, such as the death of a loved one, family conflict, or romantic abuse (Pollmann et al., 2022), rather than positive social experiences such as caregiver support, neighborhood safety, or school engagement, for example. Adverse experiences are defined as potentially traumatic events, such as abuse and neglect (Felitti et al., 1998). Adolescence is characterized by broad social experiences, including, but not limited to, those investigated in adversity research. When positive social experiences have been studied, they are usually examined in isolation, for example, investigating the relationship between friendship quality and cortical development (Becht et al., 2021;Ferschmann et al., 2019). Adolescents face many social experiences, from caregiver relationships to greater independence and exploration of the neighborhood to school pressures and engagement. Development occurs in a complex system of interacting levels of experience including individual (e.g., prosocial behaviour), interpersonal (e.g., family cohesion, number of close friends), and community (e.g., school environment, neighborhood safety) levels (Bronfenbrenner, 1979;Pollmann et al., 2025). It is, therefore, important to capture the complexity of social experiences in our investigations of adolescent development.

Accelerated cortical thinning during adolescence is associated with cognitive development, such as the development of emotion regulation (Vijayakumar et al., 2014), as well as mental health (Bos et al., 2018). Given the evidence for protracted social development in adolescence (Blakemore & Mills, 2014), it is surprising that few studies have examined how social experiences interact with cortical development during adolescence, and there are few longitudinal investigations. A study published in 2021 found that higher prosocial behavior was associated with greater cortical thinning in early adolescence (Ferschmann et al., 2019). Other studies have examined friendship quality and found a bi-directional relationship between increasing friendship quality and faster cortical thinning in the pre-frontal cortex over three bi-annual waves starting at age 14 years (Becht et al., 2021). Cross-sectional evidence from 989 participants aged 9–10 from the Adolescent Brain Cognitive Development (ABCD) sample has shown less supportive caregiving, higher family conflict, and higher perceived neighborhood safety were associated with greater cortical thickness (Hong et al., 2021). This shows the interaction between cortical thinning and social experiences is likely not limited to peer relationships but extends to adolescents’ broad social environment, including the school and neighborhood. Importantly, this study found data were best explained by clusters of multiple environmental and brain structure factors, rather than subtypes of single factors. These findings imply that cortical development may be particularly susceptible to individual differences in social experiences. Yet, existing evidence is currently limited to either single time points or individual social experiences and does not account for the complexity of adolescents’ social experiences. Multivariate, longitudinal models of social experiences that account for friend and family relationships, as well as school and neighborhood factors, are required to reflect the variety and complexity of adolescents’ social lives more accurately and to better understand how neurobiological changes in adolescence interact with the broadening social environment.

### 1.2 The current study

This study investigated how pubertal stage relates to the relationship between positive social experiences and cortical development during early adolescence. Two waves, 2 years apart, were available at the time of analyses (June 2022) from the Adolescent Brain Cognitive Development study (ABCD: N ~ 12,000,https://abcdstudy.org) at 9–11 years and 11–13 years. We fit a bivariate latent change score model to test our hypotheses. Latent change score models are a powerful method to investigate how latent factors predict the rate of change in development over time (Kievit et al., 2018). A unique facet of this model is that we can derive latent factors capturing shared variance of multiple measures of perception of social experiences and test how these latent factors predict cortical development. This allows us to model the patterns underlying adolescents’ diverse social experiences and examine how they relate to differences and changes in cortical thickness. We complemented this analysis by fitting separate indicator models to understand relationships between perceptions of individual social experiences and cortical thickness. We then fit a multi-group model to test whether the relationship between cortical thickness and positive social experiences over time differed depending on the stage of puberty of both males and females.

We hypothesized that more positive social experiences at age 9–11 would predict greater change in cortical thickness, specifically a reduction in cortical thickness, between age 9–11 and 11–13 years. We also expected to find evidence for correlated change, such that an increase in positive social experiences would be associated with a greater reduction in cortical thickness over time. Given the limited evidence for the relationship between puberty stage, cortical development, and real-life experiences (Goddings et al., 2019), we did not stipulate a priori hypotheses about the direction of effects. Still, we did hypothesize that the negative relationships between positive social experiences and cortical thickness would vary depending on the stage of puberty in males and females. Models were first developed in a randomly selected sample of 20% of the dataset (our exploratory sample; results are reported on OSF:https://osf.io/cmpnz/) and then replicated in the remaining 80% (our confirmatory sample) (Akker et al., 2021). The confirmatory sample results are presented in this article.

## 2 Materials and Methods

### 2.1 Study design

Data from baseline and the 2-year follow-up were analyzed from the ABCD sample: a longitudinal cohort where participants were 9–11 years at baseline (Casey et al., 2018;Gonzalez et al., 2021;Zucker et al., 2018). Cognitive and behavioral data are collected every year, with the addition of structural and functional magnetic resonance imaging and bioassays in alternate years. Data were downloaded for baseline and 2-year follow-up in April 2022 from ABCD Data Release 4.0. The ABCD Data Release 5.0 was published in 2023 after data analysis was conducted. ABCD report minor changes to structural MRI pre-processing that do not affect global, whole-brain measures (see here for details:https://nda.nih.gov/study.html?id=2147). A co-author, who did not conduct data analysis (AP), randomly selected 20% of the sample as the exploratory sample for us to test model convergence and to write the analysis scripts. This analysis was then replicated in the test sample (remaining 80%). This allowed us to develop models that best fit the data and then cross-validate the findings in the main sample. This approach has been recommended to control researcher degrees of freedom in studies where pre-registration is not feasible (Akker et al., 2021). The results from the confirmatory sample models did not differ from the exploratory sample (seehttps://osf.io/cmpnz/for exploratory sample results). Demographic characteristics of age, sex, puberty stage and socio-economic status did not differ between the two samples (seeSupplementary Methods 1). The test sample results are presented in the main manuscript.

### 2.2 Cohort

This study used data from the longitudinal Adolescent Brain Cognitive Development study (N ~ 12,000;https://abcdstudy.org/). We used data collected at baseline (aged 9–11 years) and at 2-year follow-up (aged 11–13 years). The confirmatory sample included N = 9,501 (48% females, 52% males; baseline age: M = 9.91, SD = .63; 2-year-follow-up age: M = 12.01, SD = .66). Participant ethnicity was categorized by ABCD as White, Black, Hispanic, Asian, and Other. Over half of the participants (52%, N = 4,924) identified their ethnicity as white, 15% (N = 1,459) as Black, 20% (N = 1,923) as Hispanic, 2% as Asian (N = 206), and 10% (N = 987) were categorized into the “Other” category. Exclusions are detailed under each measure in the methods section. The percentage of missingness ranged from 0.14% to 37.76% per variable; the number of participants missing and the percentage of participants missing per variable are presented inSupplementary Table 1. Participants and their families gave full informed consent. A detailed explanation of the ethical procedures in the ABCD study can be found inClark et al. (2018).

### 2.3 Measures

Descriptive statistics for all measures per stage of puberty are presented inSupplementary Tables 2A-D.

#### 2.3.1 Stage of puberty

The stage of puberty categorical variable was extracted from the parent-reported Pubertal Developmental Scale (PDS) (Petersen et al., 1988). Parents rated the participant’s physical development on several characteristics, including height, body hair, and skin (for both girls and boys), menarche onset and breast development (for girls), and facial hair and voice deepening (for boys). Items were rated on a four-point Likert scale from “had not begun” to “already complete”. Parent reports were used here based on suggestions that they are more accurate than youth reports in early adolescent samples (Cheng et al., 2021). Here, we used the ABCD-derived categories from the PDS. The categorical score consists of five pubertal stages: pre-puberty, early, mid, late, and post-puberty. We stratified by sex and puberty status into four groups: male early (early puberty stage), female early, male non-early stage (pre-, mid-, late-, post-puberty), and female non-early stage, to maximize power and aid convergence and because we were primarily interested in the early stage as this is when there is the greatest rate of change in puberty hormones. As a robustness check suggested by a reviewer, we conducted a posthoc analysis, where we regrouped into consecutive groups: male early (pre-, early stage), female early, male late (mid-, late-, post-puberty) and female late, and refit the multi-group model to compare between groups. The findings did not differ and are presented in theSupplementary Results 3. This procedure is explained in more detail inSection 2.4.

#### 2.3.2 Positive social experience measures

We captured perceived positive social experiences via self-reported measures of experiences among peers (prosocial behavior and number of close friends), among family (caregiver monitoring, family cohesion), and among the broader environments of school (school environment, school involvement) and neighborhood (neighborhood safety). We were interested in how young people perceive social experiences, how that might change over time, and in developing a model accounting for a broad set of social experiences. Our model of social experiences is designed to capture the variety of experiences in adolescents’ social lives at multiple levels: individual (e.g., prosocial behavior), interpersonal (e.g., family cohesion, number of close friends), and community (e.g., school environment, neighborhood safety) levels. This aligns with Bronfenbrenner’s ecological systems theory that multiple levels of systems in the environment interact with one another to shape human development (Bronfenbrenner, 1979). We use the term*perception of social experiences*to capture the nature of the measures used in ABCD. These capture how participants feel about their social experiences, rather than the number of social experiences, for example. Detailed descriptions of the measures are provided elsewhere (Gonzalez et al., 2021;Zucker et al., 2018). Variables described below were computed by ABCD, and variable names are provided inSupplementary Tables 2A-Dto aid replication.

The prosocial behavior variable is the mean score of three prosocial items from the Strength and Difficulties Questionnaire Prosocial Scale (Goodman, 1997;Goodman et al., 1998), for example, “I try to be nice to other people. I care about their feelings”. Responses were from Not True to Certainly True (0 to 2), and a minimum of two answers was required for the mean variable. For number of close friends, participants reported the number of close (best) friends they had, friends that they “like spending time with, have fun with, and trust” (values greater than 100 were truncated at 100, and the variable was summed across sex of friends).

The School Risk and Protective Factors (SRPF) scale is made up of School Environment, School Involvement, and School Disengagement scales and is a subset of the PhenX SPRF designed to capture young people’s perceptions of school (Arthur et al., 2007;Zucker et al., 2018). As we were interested in positive social experiences, we did not include school disengagement. The School Environment scale consists of 6 items reflecting youth’s perception of the school environment, for example, “My teacher(s) notices when I am doing a good job and lets me know about it.” The School Involvement scale consists of 4 items indicating positive involvement in school, for example, “In general, I like school a lot.” Responses ranged from NO! to YES! (1 to 4), with a minimum of 5 answers for the School Environment scale and a minimum of 3 answers for the School Involvement scale required.

Caregiver monitoring includes five items capturing youth perception of how they are monitored by their parents, for example, “How often do your parents/guardians know where you are?”. Responses range from “Never” to “Always or Almost always” (1 to 5), and the variable was a mean of 5 items (no minimum required). In the absence of a family cohesion variable, two cohesion items from the Moos Family Environment Scale (Moos, 1990) were reverse scored so that 1 = True and 0 = False and higher scores indicated greater family cohesion. The two items included were: “If there’s a disagreement in our family, we try hard to smooth things over and keep the peace” and “In our family, we believe you don’t ever get anywhere by raising your voice.” One item from the PhenX Safety from Crime scale was administered to youth: “My neighborhood is safe from crime.” Responses ranged from “strongly disagree” to “strongly agree” (1 to 5). For all positive social experiences, participants were excluded if their z scores were +/- 5 from the mean. This criterion was only met in caregiver monitoring and number of close friends. It applied to the following Ns for caregiving monitoring (baseline: N = 2, 2-year-follow-up: N = 7) and for number of close friends (baseline: N = 72, 2-year-follow-up: N = 61).

#### 2.3.3 Cortical thickness

Mean whole-brain cortical thickness was extracted from ABCD and formed the observed variable of cortical thickness at baseline and 2-year follow-up (seeSupplementary Tables 2A-Dfor descriptive statistics). We have analyzed global cortical thickness here to allow for general developmental trends and avoid multiple comparisons in the absence of clear regional hypotheses. As cortical thickness tends to show a substantial correlation across the brain (Fjell et al., 2015), using a whole-brain approach at this stage also affords a high degree of power. Before exclusion, structural MRI data was available for 11,801 participants at baseline and 7,857 participants at 2-year follow-up. The cortical surface was reconstructed from T1w and T2w images in the ABCD data (Hagler et al., 2019). Guidelines on quality control and manual review determined exclusion criteria (see ABCD Release Notes for detailed information:https://nda.nih.gov/abcd/). Structural MRI cortical reconstruction was assessed by ABCD reviewers and assigned an overall quality control score. Participants recommended for exclusion were excluded based on this score (baseline N = 1,062 [8.98% of sample], 2-year-follow-up N = 507 [4.28% of sample]). Images were also assessed for suspected clinical issues. Participants with a score over 2 (3 = consider clinical referral, 4 = consider immediate clinical referral) were excluded (baseline N = 451 [3.79% of sample], 2-year-follow-up N = 360 [3.03% of sample]). Lastly, we checked for outliers using*z*-scores and applied an exclusion criterion of +/- 5 (no exclusions were made). This outlier cut-off is in line with previous research (Fuhrmann et al., 2021). It is designed to catch extreme outliers that could be data entry or processing errors while retaining infrequent data points that may reflect natural variation in developmental variables.

#### 2.3.4 Socio-economic status

Socio-economic status (SES) was included as a sensitivity check to test whether our results were consistent across subgroups. SES was captured using parental education, a reliable indicator in this age group (Galobardes et al., 2006;Walsh et al., 2019). While other measures of SES exist, exploring multiple indicators is beyond the scope of this paper and has been investigated elsewhere (seeRakesh & Whittle, 2021for review). Parents were asked: “What is the highest grade or level of school you have completed or the highest degree you have received?” and scores ranged from 1 = “Never attended/kindergarten only” to 21 = “Doctoral degree.” We converted the categorical score to parent education in years (as inRakesh, Zalesky et al., 2021) and divided it into high and low SES using a median split (median = 16 years). The number and percentage of participants per group are reported inSupplementary Table 3.

### 2.4 Data analysis procedure

Our main analysis scripts are provided open-access and can be downloaded from:https://osf.io/cmpnz/. Models were fit using the*lavaan*package (Rosseel et al., 2023) for R software (R Core Team, 2021). Model fit was estimated using maximum likelihood estimation with robust standard errors, and full information maximum likelihood estimation was used to model missing data.

Note that only a subset of participants (N=7,857) had complete structural MRI data at time 2. Full information maximum likelihood (FIML) was used to handle missing data. It is advantageous for handling missing data in longitudinal models because it uses all available information across both time points without imputing values, thus maximizing statistical power and reducing bias. Unlike traditional methods like listwise deletion, FIML provides unbiased estimates under the assumption that data are missing at random (Enders & Bandalos, 2001), allowing us to retain participants with incomplete data and minimizing the impact of attrition on model validity. Simulation studies have shown FIML performs better than listwise deletion in terms of parameter estimate bias, parameter estimate efficiency, goodness of fit and convergence (Enders & Bandalos, 2001). Incorporating those with missing data yields more precise estimates of the intercept and ensures more representative estimates of change.

Good fit was determined by the following indices (Schermelleh-Engel et al., 2003): reporting the*χ*^2^test, the comparative fit index (CFI; good fit = >.97, acceptable fit = .95-.97), the standardized root mean square residual (SRMR; good fit = <.05, acceptable fit = .05-.10), and the root mean square error of approximation (RMSEA; good fit = <.05, acceptable fit = .05-.08). For measurement invariance tests, the following criteria were used to compare nested models: a non-significant chi-squared test (*p*> .05), <.015 increase in RMSEA, and/or < .010 decrease in CFI warranted a more stringent model (e.g., from weak to strong invariance) (Luong & Flake, 2023).

We used a multi-step multivariate approach to test how puberty stage relates to the relationship between positive social experiences and cortical thickness. We leveraged a latent variable model of social experiences as a pragmatic way of modelling covariance between individual indicators of social experiences. To examine the measurement consistency of the social experiences variables over time, we first fit a longitudinal confirmatory factor analysis (CFA). Longitudinal CFA enables us to assess the stability and validity of the seven positive social experiences (prosocial behavior, number of close friends, school environment, school involvement, caregiver monitoring, family cohesion, and neighborhood safety) across the baseline and 2-year follow-up. This step ensures that the same construct is consistently measured over time, reducing measurement error, and strengthening the reliability of the latent variable.

Next, we fit a bivariate latent change score model (LCSM) with positive social experiences as a latent factor and cortical thickness as an observed variable. The bivariate LCSM allows us to model changes in each construct over time and tests the temporal relationships between the rate of change in positive social experiences and the rate of change in cortical thickness. This dynamic approach captures potential co-development or directional influences, revealing how shifts in one domain may relate to changes in the other across the two time points of early adolescence. Variables were first scaled to values of 0-100 to facilitate model convergence.

To complement the model capturing shared variance of the range of positive social experiences, we investigated the relationship between social experiences and cortical thickness in separate indicator models—in other words, examining each individual variable reflecting social experiences in turn. This approach allows us to assess the unique contribution of each specific aspect of social experience, providing insight into which individual factors may drive the observed associations. These results are presented in theSupplementary Results 2.

To investigate whether there were differences in the relationship between positive social experiences and cortical thickness between stages of puberty for males and females, we compared a model where our parameter of interest determined by the results from the bivariate latent change score model was allowed to vary between groups, to a second model where this parameter interest was constrained between groups. In all multi-group models, we stratified for sex and puberty stage. Recommendations from the literature have suggested to derive three groups from the PDS categorical score pre-/early-puberty, mid-puberty, and late-/post-puberty (Koopman-Verhoeff et al., 2020). Once stratified by sex, this created six groups and a model with 354 parameters, which led to model convergence issues. To maximize power and ensure model convergence, we contrasted the early puberty stage with all non-early groups (pre-puberty, mid-puberty, late puberty, and post-puberty) for males and females (seeTable 1for sample sizes) given that the early puberty stage involves the highest rate of change in puberty hormones (Mendle et al., 2019). Because our non-early puberty stage group contained non-consecutive puberty stages, for example, pre-puberty and late puberty, we ran a second multi-group analysis where participants were grouped into four groups by sex and consecutive puberty stages: early (pre-puberty and early puberty) and late (mid-puberty, late puberty, post-puberty) for males and females as a post-hoc robustness check. The main findings from this analysis did not differ from our initial multi-group model. We have reported the post-hoc analysis results with consecutive puberty stage groupings in theSupplementary Results 3.

**Table 1.** Number and percentage of participants in early stage and non-early puberty stage groups.

Puberty stage group	Number of participants	Percentage of sample (%)
Female		
Early	1025	11
Non-early	3331	35
Missing	168	3.7
Male		
Early	1143	12
Non-early	3625	38
Missing	176	3.5

Previous research has found that young people from low SES backgrounds experienced earlier puberty onset and lower cognitive outcomes than their high SES counterparts (Stumper et al., 2020). To check our results were consistent over SES subgroups, we split participants into high and low socio-economic status (SES) using a median split in parental education in years. We fit a multi-group model to test for possible differences in the relationship between social experiences and cortical thickness in high versus low SES.

## 3 Results

### 3.1 Positive social experiences measurement model and longitudinal invariance testing

We captured perceived positive social experiences via self-reported measures of experiences among peers (prosocial behavior, number of close friends), among family (caregiver monitoring, family cohesion), and among the broader environments of school (school environment, school involvement) and neighborhood (neighborhood safety). We were interested in how adolescents perceive social experiences, how that might change over time, and developing a model accounting for a broad set of social experiences. We fit a longitudinal confirmatory factor analysis to identify shared variance in these variables.

#### 3.1.1 Longitudinal invariance testing

To test whether the confirmatory factor analysis model fit across time points, we conducted longitudinal measurement invariance tests. In invariance testing, models with different stringencies are fit to examine whether the measurement model is consistent (in this case) across time. A*configural invariance*model was fit first, which allows the same indicators across groups but permits factor loadings and intercepts to vary. Next, we tested*weak invariance*, where factor loadings were constrained to be equal over time, followed by*strong invariance*, where both factor loadings and intercepts were constrained to be equal. A partial invariance model showed excellent fit (*χ*^2^(79) = 1309.31,*p*< .001; RMSEA = .040 [.039-.042]; CFI = .941; SRMR = .037), and the CFI increased going from partial to strong invariance constraints (ΔCFI = .037; seeSupplementary Table 4for the full set of fit statistics), suggesting that the partial invariance model approximates the data reasonably well. Factor loadings and covariances are presented inFigure 1. Some factor loadings were relatively low and below the recommended loading of .3, for example, at time 1: number of close friends = .10, neighborhood safety = .20, family cohesion = .26 (Knekta et al., 2019). These indicators were retained as they were of theoretical importance for the model: Evidence shows number of friends, neighborhood safety, and family cohesion are each associated with adolescent neurodevelopment (Rakesh, Seguin et al., 2021;Shen et al., 2023;Stepanous et al., 2023) and are important elements of adolescents’ social lives. As a robustness check, we fit the bivariate latent change score model with a reduced factor structure where indicators with loadings <.3 were removed (neighborhood safety, family cohesion, number of close friends). The model showed excellent fit (χ^2^(54) = 969.84,*p*< .001; RMSEA = .046 [.043-.048]; CFI = .957; SRMR = .040) and the results were highly similar in model fit and showed the same pattern of results (seeSupplementary Table 5).

**Fig. 1.**
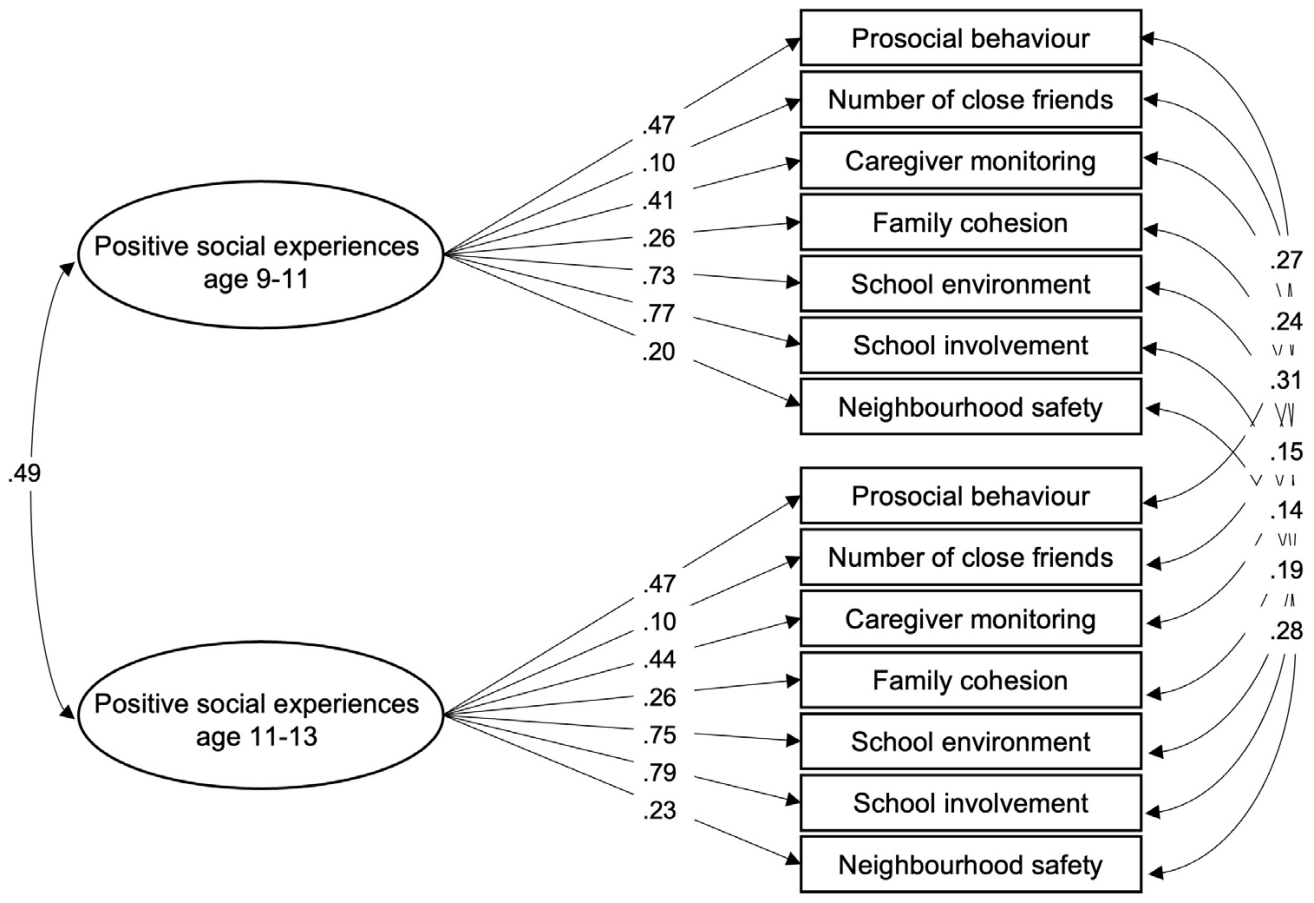
Longitudinal confirmatory factor analysis of positive social experiences at baseline (9–11 years) and 2-year-follow-up (11–13 years), equality-constrained (fully standardized) factor loadings and residual covariances across measurement occasions.

### 3.2 Evidence for correlated change between positive social experiences and cortical thickness

**Fig. 2.**
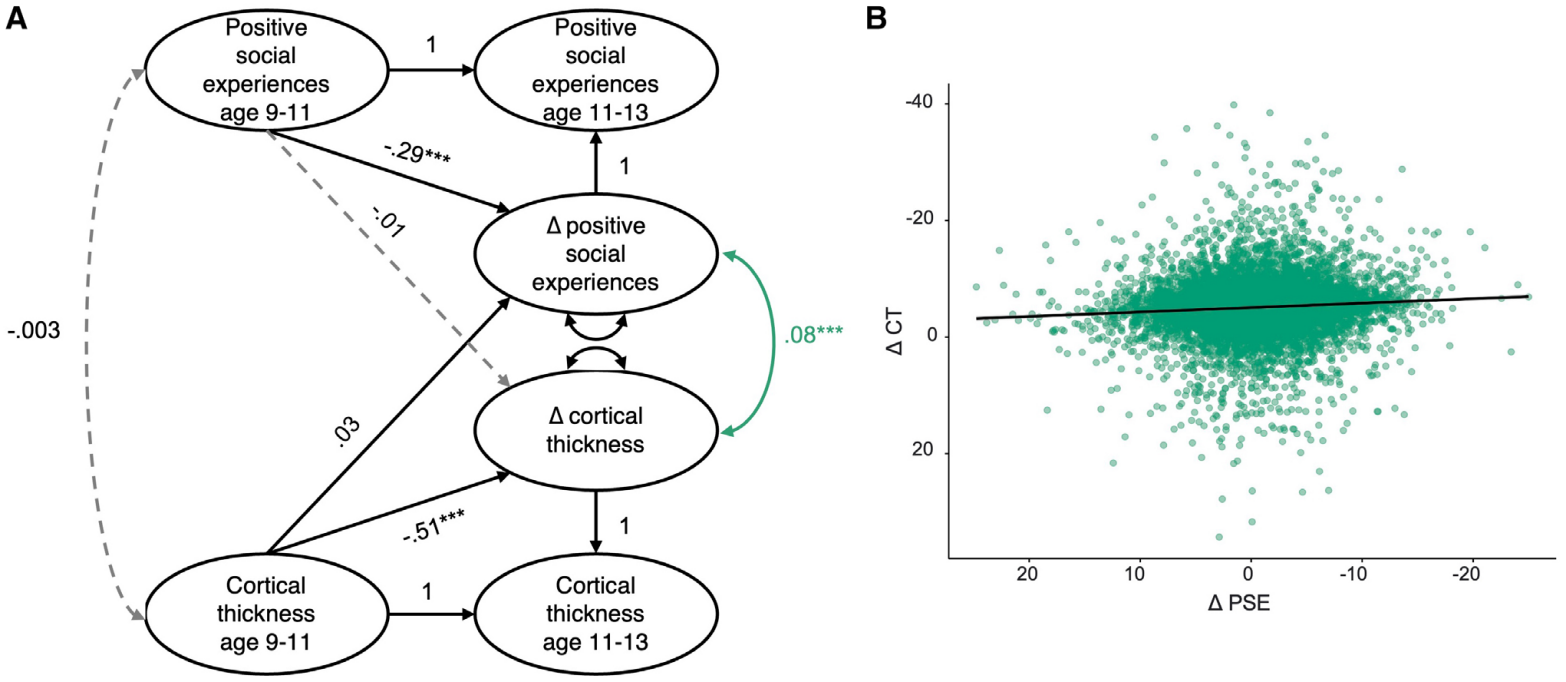
(A) Bivariate latent change score model between positive social experiences and cortical thickness at age 9–11 and 11–13 years. The full measurement model for social experiences is not shown but is the same as inFigure 1. The delta symbol (Δ) is used to indicate the latent change score, for example, “Δ positive social experiences” indicates positive social experiences change score. (B) Visualization of covariance between change in positive social experiences and change in cortical thickness (green, non-directional path in A). *** indicates significant paths after Bonferroni correction (*p*< .001), grey dashed lines indicate non-significant paths. CT = cortical thickness, PSE = positive social experiences.

**Fig. 3.**
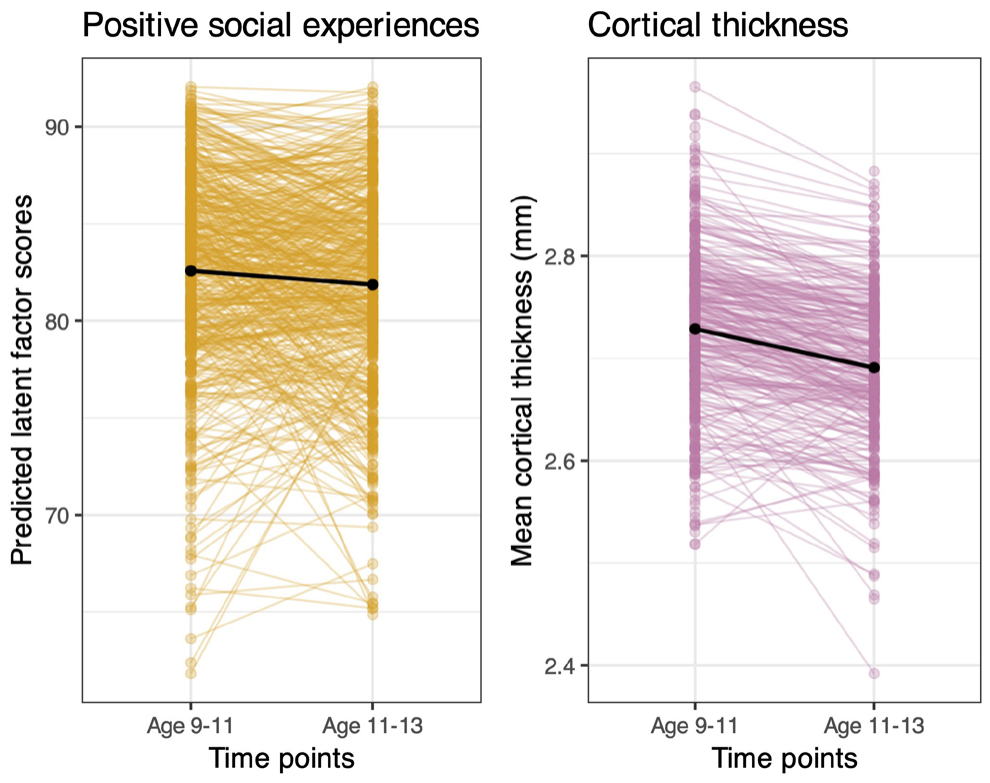
Predicted values for positive social experiences latent factor (left, yellow) and raw cortical thickness (right, pink) at both time points. Random sample of 500 participants shown for visual clarity.

We fit a multiple-indicator bivariate latent change score model (Fig. 2A) including both the latent variable for perceived positive social experiences, as determined in the first step of the analysis, as well as the observed variable cortical thickness, to examine how they co-developed across two waves. Both positive social experiences and cortical thickness decreased over time (Fig. 3). We found excellent model fit for the latent change score model: (*χ*^2^(106) = 1680.72,*p*< .001; RMSEA = .040 [.038-.041]; CFI = .943; SRMR = .039), allowing us to examine the key parameters of interest. Cortical thickness at baseline significantly predicted changes in cortical thickness (est = -.16, SE = .01, z = -18.20,*p*< .001**;**Fig. 2A). This can be interpreted as a negative self-feedback parameter, which reflects an individual’s tendency for change in cortical thickness to differ in relation to their baseline level at the first time point. There were also significant individual differences in the change in cortical thickness (est = 35.28, SE = 1.13, z = 19.66,*p*< .001), in line with evidence from previous longitudinal studies (Fuhrmann et al., 2022). Together, these results suggest that individuals with higher cortical thickness at baseline showed a more pronounced decrease in cortical thickness over time. In contrast, individuals with lower cortical thickness at baseline exhibited a relatively smaller decrease or even a slight increase over time. This pattern can be understood as a mixture of two patterns: First as a form of “regression to the mean,” where initial extreme values (high or low) tend to move closer to the average over time. Second, as a ‘true’ pattern reflecting the fact that individuals who have already (mostly) matured in terms of their cortex (i.e., have a thinner cortex) will, on average, show less thinning than those who still have to complete part or all of the developmental process (despite having the same calendar age), reflecting individual differences in the onset of thinning (cf.Fuhrmann et al., 2022). The same pattern was found in participants’ positive social experiences such that those with higher perception of social experiences at baseline showed a greater decrease in their perception of positive social experiences over time (est = -.51, SE = .02, z = -33.17,*p*< .001). Participants’ positive social experiences latent factor scores, and cortical thickness at both time points are visualized inFigure 3.

Contrary to our first hypothesis, positive social experiences at age 9–11 years did not predict change in cortical thickness over time (est = -.01, SE = .01, z = -.79,*p=*.432). However, there was evidence for correlated change in positive social experiences and change in cortical thickness, such that a greater reduction in positive social experiences was associated with a greater reduction in cortical thickness between age 9–11 years and 11–13 years (est = 2.62**,**SE = .55, z = 4.75,*p*< .001, standardized effect size = .08). This is particularly notable given that positive social experiences and cortical thickness were not significantly associated at baseline (est = -.18, SE = .89, z = -.21,*p*=.837, standardized effect size = .003). This suggests that while positive social experiences did not predict change in cortical thickness, the development of positive social experiences and cortical thickness are linked throughout early adolescence. Additional parameter estimates,*p*-values, and effect sizes are presented inTable 2and predicted values of the positive social experiences latent and raw cortical thickness are presented inFigure 3. As a complementary analysis, we fit separate indicator models, which are presented in Supplementary Results 2: the standardized effect sizes were larger in the latent variable modeling, suggesting the benefits of the latent variable model in reducing measurement error.

**Table 2.** Additional parameter estimates for the positive social experiences and cortical thickness bivariate latent change score model.

Parameter	Estimate (B)	Standard error (SE)	z-value	*p-* value	Standardized estimate
PSE change intercept	43.38	1.34	32.42	<.001	6.67
PSE latent factor intercept at baseline	80.74	.17	487.08	<.001	12.37
PSE latent change score variance	31.26	1.52	20.62	<.001	.74
Self-feedback: PSE at baseline – change in PSE between baseline and 2-year-follow-up	-.51	.02	-33.17	<.001	-.51
CT change intercept	5.16	1.25	4.13	<.001	.83
CT intercept at baseline	57.14	.12	479.21	*<* .001	5.13
CT latent change score variance	35.27	1.13	31.27	<.001	.91
Self-feedback between CT at time 1 and change in CT	-.16	.009	-18.20	<.001	-.29
Covariance between PSE change score and CT change score	2.63	.55	4.75	<.001	.08
Coupling parameter between PSE at time 1 and change in CT	-.01	.01	-.79	.432	-.01

Note: PSE = positive social experiences, CT = cortical thickness.

### 3.3 No evidence for differences in the relationship between social experiences and cortical thickness between puberty stage groups in males and females

We fit a multi-group model to investigate differences in correlated change between the rate of change in positive social experiences and rate of change in cortical thickness between participants at different stages of puberty. Participants were stratified into groups based on sex and puberty stage. To aid model convergence, participants were grouped into male early (early puberty stage), female early, and male non-early (pre-, mid-, late, post-puberty stage) as we were primarily interested in the early puberty stage. As a post-hoc robustness check, we split into consecutive groups and fit a second multi-group model: male early (pre-, early-) female early, male late (mid-, late-, post-puberty), and female late. The findings from the robustness check analysis did not differ from our a-priori analysis and are presented inSupplementary Results 3. For transparency, we have reported our a-priori grouping and primary analysis here in the main text. We found evidence for weak invariance across groups before fitting the multi-group model (seeSupplementary Results 1).

Applying the partially invariant model (seeSection 3.1for details), we tested whether there was evidence for differences between groups in the parameter of interest: the covariance between rate of change in positive social experiences and rate of change in cortical thickness. To do this, we fit models where the parameter was allowed to vary between pubertal stage group and a model where this relationship was constrained. There was no significant difference in model fit between the two models (Δ*χ*^2^(3) = 3.31,*p*= .346). This provides evidence for the more parsimonious model, suggesting no evidence for a difference in the relationship between change in positive social experiences and change in cortical thickness between pubertal stage group. To check our results were consistent across subgroups, we fit a multi-group model with high and low SES groups and there was no evidence for a significant difference in the covariance between change in cortical thickness and change in social experiences (parameter of interest). These results are presented in theSupplementary Results 4.

As an additional exploratory analysis, we examined potential mean differences in positive social experiences (as quantified by latent factor mean scores) across puberty stage groups. Females reported a slightly more positive perception of social experiences than males. Positive social experiences decreased more over time for males in the early puberty stage group compared to males in the other puberty stages (non-early); further details on the results are reported in theSupplementary Results 5.

## 4 Discussion

Using a large sample with two time points aged 9–13 years and a multivariate latent variable approach, we investigated how individual differences in the stage of puberty relate to the relationship between positive social experiences and cortical development between ages 9–11 (N = 9,501) and 11–13 years (N = 7,857) in the ABCD sample. We found evidence for individual differences in trajectories: individuals with higher scores at baseline decreased more, and individuals with lower scores at baseline increased more, in both positive social experiences and cortical thickness. Contrary to our expectations, positive social experiences at baseline did not predict changes in cortical thickness at the 2-year follow-up. We did find evidence, however, for correlated change between positive social experiences and cortical thickness, such that greater reductions in positive social experiences were associated with greater reductions in whole-brain cortical thickness over time. We did not find evidence for differences in correlated change between different puberty stage groups. We found evidence for sex-specific differences in social experiences between puberty stages: females reported social experiences as more positive than males, and males in the early puberty stage reported a greater decrease in positive social experiences over time than males in the non-early puberty stages.

Previous literature has identified a relationship between cortical thickness and individual social experiences (e.g., friendship quality;Becht et al., 2021). We adopted complementary latent variable approaches and separate indicator models that allowed us to test the relationship between cortical thickness and a range of social experiences. First, we found evidence for individual differences in the rate of change in cortical thickness. Adolescents who started with high cortical thickness showed stronger decreases in cortical thickness. This evidence supports recent longitudinal findings of individual differences in the rate of cortical thinning during early adolescence (Fuhrmann et al., 2022).

Our results highlight correlated change in that greater reductions in positive social experiences were associated with greater reductions in cortical thickness. This is in line with previous research showing that school engagement and perceptions of peer support decrease from around 11–14 years (Wang & Dishion, 2012;Way et al., 2007, p. 200). The ABCD sample tested here were aged between 9 and 13 years old; this is the time of transition from middle school to high school in the United States (around age 11). This developmental trend may not be unique to social experiences captured here. Other research has found similar trends in adolescents’ development of their sense of self and autonomy in early adolescence when adapting to a new school environment. For example, research has shown adolescents’ perceptions of themselves in relation to those around them and their environment—their self-concept—improve throughout adolescent years, except for a dip around 11 years old to mid-adolescence. This dip has previously been found to be pronounced in the academic domain (Cole et al., 2001;van der Cruijsen et al., 2023). Taken together with our research, we show that the link between cortical development and adolescents’ perception of social experiences extends beyond academic environments.

Despite evidence for correlated changes, positive social experiences at baseline did not predict the rate of change in cortical thickness as we expected. One plausible explanation is that we might not see a directional effect on change in cortical thickness until later in development, given evidence suggesting the steepest changes in cortical thickness occur around 14 years of age (Fuhrmann et al., 2022). It could also be that the developmental cascades we see from adverse experiences to later neurodevelopment (Teicher et al., 2016) are not evident in the context of positive social experiences. Instead, the evidence supports the transactional model of adolescent development, which suggests dynamic, transactional relationships between the environment, social experiences, and neurodevelopment throughout adolescence (Becht et al., 2021;Cheng et al., 2024;Pfeifer & Allen, 2021). This extends current knowledge concerning the susceptibility of the adolescent brain to the environment. Future studies with more time points later in adolescence are needed to formally test these different accounts.

We also investigated how the relationship between positive social experiences and cortical thickness differed between puberty stages in males and females. Contrary to our expectations, we found no evidence that the relationship between positive social experiences and cortical thickness is influenced by pubertal stage in males or females. It might also be important to consider different measures of puberty, such as hormones or puberty tempo (Cheng et al., 2021). It could also be that the effects of pubertal stage do not emerge until later in adolescence.

As an additional exploratory analysis, we compared positive social experiences latent factor mean scores across puberty stages. We found evidence for females perceiving social experiences as more positive than males, and males in the early stage of puberty showed a greater decrease in perception of positive social experiences over time than males in the other stages of puberty. Previous studies have found females report greater peer support at this age and steeper declines in reported peer support over time, compared to males (Way et al., 2007), which is in line with the evidence presented here. The finding suggesting perception of positive social experiences decreased more for males in the early puberty stage compared to the late puberty stage is in line with previous research investigating academic disengagement. A 2020 study examined the relationships between puberty hormones and academic disengagement in 11–13-year-olds and found that significant associations were more consistent in males (Martin et al., 2022). This is further indication of sex-specific relationships between puberty and social experiences; however, there is generally a paucity of investigation of the relationship between puberty and social experiences for males. Future research should prioritize understanding males’ experience of early puberty in the context of their social relationships.

There are some methodological limitations to consider when interpreting the results. Here we analyzed global cortical thickness in the absence of specific regional hypotheses to maximize power. Future studies should consider region-specific analyses. It is important to recognize that the effect size of the observed correlated change scores is small. Although latent change score models are a powerful method for examining developmental changes between two or more time points (Kievit et al., 2018), only two time points of repeated measures data limit conclusions regarding longitudinal development and trajectories (Parsons & McCormick, 2022). Future studies should replicate this study with more than two time points of repeated-measures data. Using a multivariate model that captured positive social experiences across family, peers, school, and neighborhood is a strength of this study. Some of these indicators did not load strongly onto the positive social experiences factor, however. While removing these indicators does not change the pattern of results reported here, future studies with a broader set of indicators are needed to interrogate the factor structure of positive social experiences in depth. Specifically, number of close friends was the only peer relationship variable available in both time points of the ABCD cohort, however, it is not indicative of friendship quality. Future studies would benefit from including variables that capture the quality and quantity of peer relationships.

In conclusion, we used a multivariate approach to investigate how the stage of puberty relates to the change in positive social experiences and cortical thickness during early adolescence. Positive social experiences at age 9–11 years did not predict change in cortical thickness between 9–11 and 11–13 years. However, we found evidence of correlated change, such that greater reductions in positive social experiences were associated with greater reductions in cortical thickness over time. We did not find evidence for differences in correlated change between early and non-early puberty stages in males and females. We found evidence for sex-specific differences in positive social experiences, which point to the importance of better understanding the male experience of early stages of puberty in the context of their social environment. Our study demonstrated the value of adopting complementary latent variable and separate indicator model approaches when investigating the link between neurobiological changes and complex social experiences during adolescence. Together the study highlights the importance of supporting adolescents through school transitions and demonstrates of using multivariate, longitudinal approaches to capture the dynamic interplay between social experiences and cortical development throughout adolescence.

## Supplementary Material

Supplementary Material

## Data Availability

Data can be accessed with permission from the Adolescent Brain Cognitive Development (ABCD) study:https://abcdstudy.org. Our main analysis scripts are provided open-access and can be downloaded from:https://osf.io/cmpnz/.
