# Supplementary material for: Perception of social experiences and cortical thickness change together throughout early adolescence: Findings from the ABCD cohort
Source: Imaging Neurosci (Camb). 2025 Jun 6;3:IMAG.a.27. doi: 10.1162/IMAG.a.27 (PMC12319868; doi:10.1162/IMAG.a.27)

Supplementary Materials:

Perception of social experiences and cortical thickness change together throughout early adolescence: findings from the ABCD cohort

Kathryn E. Bates*[1], Ayla Pollmann[1], Rogier Kievit[2], Delia Fuhrmann[1]

[1]King's College London, [2]Donder's Institute, Radboud University

*Corresponding author:

Dr Kathryn E. Bates, kathryn.2.bates@kcl.ac.uk

Supplementary Methods 1

Welch two-sample t-tests were conducted to compare the ages of participants in the exploratory sample (20% of the full sample) and confirmatory sample (80% of the full sample). The results indicated no significant difference in age at baseline ($t(3678) = .23$, $p = .817$), where the mean age in months of the exploratory sample was 119.01 months, and the confirmatory sample was 118.97 months. There was no significant difference between age in months at 2-year-follow-up ($t(3180) = -0.59$, $p = .549$), where the mean age of months in the exploratory sample was 143.95 and 144.06 in the confirmatory sample. Chi-square tests were performed to assess differences in sex, puberty stage, and socioeconomic status (SES) between the samples. For sex, the analysis yielded no significant difference, $\chi^2(1) = .237$, $p = .626$. Similarly, the results for puberty stage revealed no significant differences, $\chi^2(3) = .697$, $p = .873$. For SES (low and high groups defined in Methods), the results showed no significant differences, $\chi^2(1) = .021$, $p = .886$.

Supplementary Table 1

Missingness per variable

Variable	N missing	Percentage missing
Cortical thickness 11-13 years	3,588	37.76
Cortical thickness 9-11 years	1,238	13.03
Number of friends 11-13 years	1,227	12.91
Caregiver monitoring 11-13 years	1,178	12.40
Family cohesion 11-13 years	1,176	12.38
School environment 11-13 years	1,175	12.37
School involvement 11-13 years	1,175	12.37
Prosocial behaviour 11-13 years	1,173	12.35
Neighbourhood safety 11-13 years	1,173	12.35
Pubertal timing	377	3.97
Number of friends 9-11 years	84	0.88
Prosocial behaviour 9-11 years	23	0.24
School environment 9-11 years	17	0.18
School involvement 9-11 years	17	0.18
Neighbourhood safety 9-11 years	16	0.17
Family cohesion 9-11 years	16	0.17
Caregiver monitoring 9-11 years	15	0.16
Parental education	13	0.14

Note: Variables are listed in order of missingness

Supplementary Table 2A

Descriptive statistics for all measures for females in the early puberty stage

		Females, early pubertal stage							
		Age 9-11 years				*Age 11-13 years*			
Variable	Variable name in ABCD	Mean	SD	Min	Max	Mean	SD	Min	Max
Prosocial behaviour	pmq_y_ss_mean	87.41	16.35	0	100	89.39	15.77	0	100
Number of close friends	resiliency5b_y, resiliency6b_y	10.63	9.91	0	92.59	10.98	9.14	0	87.04
Caregiver monitoring	pmq_y_ss_mean	84.41	14.37	0	100	87.78	12.01	31.25	100
Family cohesion	psb_y_ss_mean	85.64	26.97	0	100	84.74	26.88	0	100
School environment	*srpf_y_ss_ses*	79.75	14.31	11.11	100	76.84	14.38	11.11	100
School involvement	*srpf_y_ss_iiss*	80.31	17.26	8.33	100	74.82	18.11	0	100
Neighbourhood safety	*neighborhood_crime_y*	77.1	26.12	0	100	77.76	23.89	0	100
Cortical thickness	*smri_thick_cdk_mean*	2.74	0.07	2.45	2.97	2.69	0.07	2.48	2.94
Parental education	*demo_prnt_ed_v2*	81.08	10.45	44.44	100	N/A	N/A	N/A	N/A

Note: Parental education was only collected at baseline and the mean was taken after we converted the variable to years

Supplementary Table 2B

Descriptive statistics for all measures for females in the non-early puberty stages

		Females, non-early puberty stages							
		Age 9-11 years				Age 11-13 years			
Variable	Variable name in ABCD	Mean	SD	Min	Max	Mean	SD	Min	Max
Prosocial behaviour	pmq_y_ss_mean	87.43	16.75	0	100	88.01	17.02	0	100
Number of close friends	resiliency5b_y, resiliency6b_y	11.08	10.85	0	98.15	10.94	9.16	0	77.78
Caregiver monitoring	pmq_y_ss_mean	83.63	14.71	12.5	100	85.81	13.46	12.5	100
Family cohesion	psb_y_ss_mean	84.22	27.26	0	100	81.84	29.33	0	100
School environment	*srpf_y_ss_ses*	78.92	15.15	5.56	100	76.49	15.39	0	100
School involvement	*srpf_y_ss_iiss*	78.14	19.01	0	100	72.96	19.69	0	100
Neighbourhood safety	*neighborhood_crime_y*	75.4	27.01	0	100	76.63	25.02	0	100
Cortical thickness	*smri_thick_cdk_mean*	2.73	0.08	2.35	3.01	2.69	0.08	2.41	2.95
Parental education	*demo_prnt_ed_v2*	80.64	11.36	44.44	100	N/A	N/A	N/A	N/A

Note: Parental education was only collected at baseline and the mean was taken after we converted the variable to years

Supplementary Table 2C

Descriptive statistics for all measures for males in the early puberty stage

		Males, early puberty stage							
		Age 9-11 years				*Age 11-13 years*			
Variable	Variable name in ABCD	Mean	SD	Min	Max	Mean	SD	Min	Max
Prosocial behaviour	pmq_y_ss_mean	79.94	19.73	16.67	100	82.53	19.75	0	100
Number of close friends	resiliency5b_y, resiliency6b_y	10.9	11.64	0	100	11.82	11.11	0	85.19
Caregiver monitoring	pmq_y_ss_mean	78.23	17.3	6.25	100	81.85	15.23	18.75	100
Family cohesion	psb_y_ss_mean	82.57	27.98	0	100	81.4	28.74	0	100
School environment	*srpf_y_ss_ses*	75	16.91	0	100	74.05	16.09	0	100
School involvement	*srpf_y_ss_iiss*	72.83	20.5	0	100	69.55	20.46	0	100
Neighbourhood safety	*neighborhood_crime_y*	73.79	28.24	0	100	75.57	26.41	0	100
Cortical thickness	*smri_thick_cdk_mean*	2.71	0.08	2.38	2.96	2.68	0.08	2.32	2.91
Parental education	*demo_prnt_ed_v2*	81.28	11.29	44.44	100	N/A	N/A	N/A	N/A

Note: Parental education was only collected at baseline and the mean was taken after we converted the variable to years

Supplementary Table 2D

Descriptive statistics for all measures for males in the non-early puberty stages

		Males, non-early puberty stages							
		Age 9-11 years				Age 11-13 years			
Variable	Variable name in ABCD	Mean	SD	Min	Max	Mean	SD	Min	Max
Prosocial behaviour	pmq_y_ss_mean	80.72	19.65	0	100	82.73	19.34	0	100
Number of close friends	resiliency5b_y, resiliency6b_y	10.39	10.69	0	96.3	11.06	10.56	0	87.04
Caregiver monitoring	pmq_y_ss_mean	78.19	16.47	0	100	82.43	14.69	12.5	100
Family cohesion	psb_y_ss_mean	81.59	28.53	0	100	82.65	28.16	0	100
School environment	*srpf_y_ss_ses*	76.23	15.69	0	100	75.49	15.25	0	100
School involvement	*srpf_y_ss_iiss*	72.49	20.08	0	100	71.61	18.95	0	100
Neighbourhood safety	*neighborhood_crime_y*	76.8	26.97	0	100	78.54	24.21	0	100
Cortical thickness	*smri_thick_cdk_mean*	2.73	0.08	2.47	3.00	2.70	0.08	2.39	3.03
Parental education	*demo_prnt_ed_v2*	81.03	10.57	44.44	100	N/A	N/A	N/A	N/A

Note: Parental education was only collected at baseline and the mean was taken after we converted the variable to years

Supplementary Table 3

Number and percentage of participants in each SES category

Parent education at baseline	N	Percentage
Third grade	-	-
Fourth grade	-	-
Fifth grade	-	-
Sixth grade	47	0.49
Seventh grade	16	0.17
Eighth grade	50	0.53
Ninth grade	101	1.06
Tenth grade	88	0.93
Eleventh grade	158	1.66
Twelfth grade	152	1.60
High school graduate	824	8.67
GED or equivalent	224	2.36
Some college	1565	16.47
Associate degree: Occupational	698	7.35
Associate degree: Academic program	533	5.61
Bacheler's degree (e.g., BA)	2623	27.61
Master's degree (e.g., MA)	1824	19.20
Professional school degree (e.g., MD)	257	2.70
Doctoral degree	311	3.27
Missing	-	-

Note: Values below 10 have been removed to prevent statistical disclosure

Supplementary Table 4

Model fit for configural, weak, partial and strong invariance for longitudinal confirmatory factor analysis models

**Model**	**χ^2(df)**	***p* value**	**RMSEA (lower 90% CI, upper 90% CI)**	**ΔRMSEA**	**CFI**	**ΔCFI**	**SRMR**	**$\Delta\chi^2$(Δdf), *p***
Configural	1237.23 (69)	< .001	.042 (.040, .044)	-	.944	-	.036	-
Weak	1240.34 (75)	< .001	.040 (.038, .042)	.002	.944	.000	.036	2.62(6), .854
Partial	1309.31 (79)	< .001	.040 (.039, .042)	.011	.941	.003	.037	69.20(4), <.001
Strong	2087.19 (82)	< .001	.051 (.049, .053)	.011	.904	.037	.042	735.64(3), <.001

Note: Partial invariance here indicates a strong invariant model where caregiver monitoring, school environment and school involvement were allowed to vary over time.

Supplementary Table 5

Parameter estimates for a reduced bivariate latent change score model with the three lowest factor loadings removed (number of close friends, family cohesion, neighbourhood safety)

**Parameter**	**Estimate (B)**	**Standard error (SE)**	**z-value**	***p*-value**	**Standardized estimate**
PSE change intercept	43.93	1.34	32.81	< .001	6.86
PSE latent factor intercept at baseline	80.74	.17	487.09	< .001	12.64
PSE latent change score variance	30.24	1.49	20.32	< .001	.74
Self-feedback: PSE at baseline – change in PSE between baseline and 2-year-follow-up	-.51	.02	-33.46	< .001	-.51
CT change intercept	5.42	1.27	4.27	< .001	.87
CT intercept at baseline	57.14	.12	479.23	< .001	5.13
CT latent change score variance	35.24	1.28	31.27	< .001	.91
Self-feedback between CT at time 1 and change in CT	-.16	.009	-18.20	< .001	-.29
Covariance between PSE change score and CT change score	2.59	.54	4.80	< .001	.08
Coupling parameter between PSE at time 1 and change in CT	-.01	.01	-.99	.321	-.02

Note: PSE = positive social experiences, CT = cortical thickn

Supplementary Results 1

Invariance models were fit with groups stratified by sex and puberty stage. We started with *configural invariance,* which allows the same indicators across groups but permits factor loadings and intercepts to vary. Next, we tested *weak invariance*, where factor loadings were constrained to be equal across groups, followed by *strong invariance*, where both factor loadings and intercepts were constrained. Testing these models helps us determine whether the factor structure and intercepts are comparable across groups before examining group differences in our parameter of interest. We considered delta RMSEA, delta CFI and the chi-squared test results in determining the best-fitting model (as in the longitudinal invariance procedure and recommended in previous literature; Luong & Flake, 2023). In comparing the weak vs. partial invariance models (neighbourhood safety was allowed to vary in the latter), the chi-square and CFI test results suggested retaining a weak invariance model ($p < .001$), with an inconclusive ΔCFI = .013), while the difference in RMSEA was negligible (ΔRMSEA = .003; see Supplementary Table 6). On balance, we decided to retain the weak invariance model. We also checked the measurement invariance for sex (males and females). Chi-squared tests were significant between each of the models, th ande difference in RMSEA was negligible, suggesting evidence for strong invariance (see Supplementary Table 6).

Supplementary Table 6

Model fit for configural, weak, and strong invariance multi-group latent change score models for puberty stage groups (early and non-early) from multi-group model

Model	χ^2(df)	*p* value	RMSEA (lower 90% CI, upper 90% CI)	ΔRMSEA	CFI	ΔCFI	SRMR	$\Delta\chi^2$(Δdf), *p*
Configural	2570.04 (448)	< .001	.046 (.044, .047)	-	.918	-	.049	-
Weak	3302.05 (487)	< .001	.050 (.049, .052)	.016	.891	.027	.056	436.16(6), <.001
Partial	3719.06 (502)	< .001	.053 (.051, .055)	.003	.875	.016	.058	424.32(15), <.001
Strong	3761.86 (505)	< .001	.053 (.052, .055)	0	.874	.001	.059	41.90(3), <.001

Supplementary Table 7

Model fit for configural, weak, and strong invariance multi-group latent change score models for sex (male and female)

Model	χ^2(df)	*p* value	RMSEA (lower 90% CI, upper 90% CI)	ΔRMSEA	CFI	ΔCFI	SRMR	$\Delta\chi^2$(Δdf), *p*
Configural	2411.90 (231)	< .001	.045 (.044, .047)	-	.916	-		-
Weak	2951.20 (237)	< .001	.050 (.049, .052)	.015	.895	.021	.050	505.21(6), <.001
Strong	3372.70 (243)	< .001	.053 (.052, .055)	.003	.879	.016	.056	430.37(6), <.001

Supplementary Results 2

We fit separate indicator latent change score models to each of the social experience indicators. We found similar patterns as in the bivariate latent change score model: there was evidence for an association between a reduction in perceived positivity of the social experience and cortical thickness for school environment (est = .75, SE = .22, z = 3.43, *p* = .001) school involvement (est = 6.37, SE = 1.46, z = 4.36, *p* < .001) and prosocial behaviour (est = .08, SE = .03, z = .286, *p* = .004). Standardised effects indicate a slightly larger effect for the covariance between change in the latent factor model (.08) compared to school involvement (.06), school environment (.05), and prosocial behaviour (.04) suggesting the benefit of the latent variable approach in reducing measurement error. Parameter estimates, *p*-values, and effect sizes for separate indicator models are presented in Supplementary Table 8.

Supplementary Table 8

Parameter estimates for separate indicator, bivariate latent change score models of social experiences and cortical thickness

Parameter	Estimate (B)	Standard error (SE)	Z value	*P* value	Standardised estimate
Prosocial behaviour (PB)					
Latent change score intercept	1.08	.03	37.18	< .001	2.55
Latent change score variance	.12	.002	51.91	< .001	.67
Covariance between PB and CT latent change scores	.08	.03	2.86	.004	.04
Self-feedback: PB at baseline – change in PB between baseline and 2-year-follow-up	-0.65	0.01	-54.97	< .001	-.58
Number of close friends (NCF)					
Latent change score intercept	5.82	.32	17.95	< .001	.84
Latent change score variance	27.53	1.10	24.93	< .001	.58
Covariance between NCF and CT latent change scores	-.67	.47	-1.43	.152	-.02
Self-feedback: NCF at baseline – change in NCF between baseline and 2-year-follow-up	-0.77	0.02	-48.76	< .001	-.65
Caregiver monitoring (CM)					
Latent change score intercept	2.97	.05	54.21	< .001	5.42
Latent change score variance	.18	.004	46.75	< .001	.60

Covariance between CM and CT latent change scores	.07	.04	1.84	.066	.03
Self-feedback: CM at baseline – change in CM between baseline and 2-year-follow-up	-0.67	0.01	-63.33	< .001	-.63
Family cohesion (FC)					
Latent change score intercept	1.25	.04	30.07	< .001	1.72
Latent change score variance	.32	.01	52.87	< .001	.61
Covariance between FC and CT latent change scores	.07	.05	1.54	.125	.02
Self-feedback: FC at baseline – change in FC between baseline and 2-year-follow-up	-0.81	0.01	-65.80	< .001	-.63
School environment (SE)					
Latent change score intercept	13.65	.29	47.49	< .001	4.12
Latent change score variance	6.98	.13	55.69	< .001	.64
Covariance between SE and CT latent change scores	.75	.22	3.43	.001	.05
Self-feedback: SE at baseline – change in SE between baseline and 2-year-follow-up	-0.71	0.01	-60.81	< .001	-.60
School involvement (SI)					
Latent change score intercept	42.62	1.39	30.58	< .001	1.94
Latent change score variance	324.39	5.39	60.19	< .001	.67
Covariance between SI and CT latent change scores	6.37	1.46	4.36	< .001	.06
Self-feedback: SI at baseline – change in SI between baseline and 2-year-follow-up	-0.63	0.01	-56.66	< .001	-.57
Neighbourhood safety (NS)					
Latent change score intercept	2.71	.07	36.74	<.001	2.18
Latent change score variance	.89	.02	53.44	< .001	.58
Covariance between NS and CT latent change scores	.17	.08	2.05	.041	.03
Self-feedback: NS at baseline – change in NS between baseline and 2-year-follow-up	-.73	0.01	-66.35	< .001	-.65

Supplementary Results 3

As described in the data analysis procedure (Section 2.3.1), we fit a second multi-group model with consecutive puberty groups as a robustness check. The early groups included those in the pre-puberty and early puberty stages and the late group included those in the mid-puberty, late-puberty and post-puberty stages, sample descriptives are reported in Supplementary Table 9.

Supplementary Table 9

Number and percentage of participants in early stage and late stage puberty groups

Puberty stage group	Number of participants	Percentage of sample (%)
	Female	
Early	2363	52.1
Late	2002	44.2
Missing	168	3.7
	Male	
Early	4494	90.5
Late	298	6.00
Missing	176	3.5

We first examined measurement invariance across groups. The chi-squared ratio test, delta RMSEA and delta CFI showed the weak model, where factor loadings and intercepts were freely estimated between groups, fit best (see Supplementary Table 10A for chi-squared tests and fit statistics). It has recently been recommended that applying measurement invariance to latent variable model group comparisons is too stringent and that potential issues should be examined rather than ruled out (Robitzsch & Lüdtke, 2023). Inspection of the factor loadings from the configural model revealed no substantial differences in the pattern of factor loadings between groups (see Supplementary Table 10B), we therefore moved forward with the weak invariant model.

In the next step, we compared a model where the key parameter (correlated change) was freely estimated across the four subgroups, to a model where it was equality constrained. There was no significant difference between the freed and constrained model fit ($\Delta\chi^2(3) = 1.38$, $p = .711$), indicating that allowing the parameter of interest to vary between groups did not fit better than the constrained, we therefore did not find evidence for differences between early and late puberty stage groups.

Supplementary Table 10A

Model fit for configural, weak, and strong invariance multi-group latent change score models for puberty stage groups for multi-group analysis with consecutive puberty stages

**Model**	**χ^2(df)**	***p* value**	**RMSEA (lower 90% CI, upper 90% CI)**	**ΔRMSEA**	**CFI**	**ΔCFI**	**SRMR**	**$\Delta\chi^2$(Δdf), *p***
Configural	3330.46 (469)	< .001	.052	-	.889	-	.054	-
**Weak**	3449.91 (487)	< .001	.052	.000	.885	.004	.058	89.84 (18), <.001
Strong	4029.95 (505)	< .001	.055	.003	.863	.022	.061	550.91 (18), <.001

Supplementary Table 10B

Factor loadings per stage of puberty group (consecutive stages) from the configural invariance model

Observed variables	Early, female	Early, male	Late, female	Late, male
		Baseline		
Caregiver monitoring	-	-	-	-
Prosocial behavior	.393	.462	.454	.519
School environment	.690	.758	.750	.804
School involvement	.728	.792	.785	.834
Number of close friends	.089	.108	.106	.126
Neighborhood safety	.182	.220	.215	.254
Family cohesion	.218	.263	.258	.303

Note: Caregiver monitoring is constrained to 1.

We then conducted the same exploratory analysis as in the main results where we investigated potential group differences in social experiences (as quantified by latent factor mean score). When we compared a model with constrained latent factor means to a model where latent factor means were able to vary between groups in the consecutive puberty stage groups, there were model convergence issues likely due to instability in parameter estimates between unequal sample sizes, specifically the late-stage male group only included 6% of the sample. The model fit for constrained ($\chi^2(511) = 8276.63$, $p < .001$; RMSEA = .081 [.080-.083]; CFI = .698; SRMR = .088) and freed ($\chi^2(508) = 10875.18$, $p < .001$; RMSEA = .094 [.093-.096]; CFI = .597; SRMR = .108) models was poor, and it was not possible to compare the models.

Supplementary Results 4

We fit a multi-group model to test whether the relationship between change in cortical thickness and change in positive social experiences was consistent across SES subgroups (high and low SES were defined by a median split in years in education). We first checked measurement invariance across groups. There was no significant difference between the configural and weak invariance models ($\Delta\chi^2(6) = 7.87$, $p = .248$) suggesting the factor loadings were not meaningfully different. Next, we compared this model (with equality constrained factor loadings) under two scenarios: One where the covariance between change parameter was allowed to vary across SES groups, and another where this parameter was constrained to be equal across groups. There was no significant difference between the two models ($\Delta\chi^2(1) = .07$, $p = .788$). This suggests that the more parsimonious model, which assumes correlated change does not differ as a function of SES, is to be preferred.

Supplementary Results 5

As an additional exploratory analysis, we examined potential mean differences in positive social experiences (as quantified by latent factor mean scores) across puberty stage groups. In other words, this analysis allows us to test whether a model that assumes social experiences were rated equally positive across the four groups did better than a model that assumes the same mean for each group. We found evidence for differences in the positive social experiences factor score across groups (chi-squared test: $\Delta\chi^2(3) = 275.58$, $p < .001$, freed model fit: $\chi^2(508) = 3765.58$, $p < .001$; RMSEA = .053 [.051-.055]; CFI = .874; SRMR = .059). Females report a slightly more positive perception of social experiences than males. Positive social experiences decreased more over time for males in the early puberty stage group, compared to males in the other puberty stages (non-early), see Figure S1. These results suggest evidence for sex-specific differences in social experiences at different puberty stages.

Figure S1. Mean and standard error for positive social experiences latent factor at baseline and year 2 for each pubertal stage group (female early stage, male early stage, female other stage, male other stage).

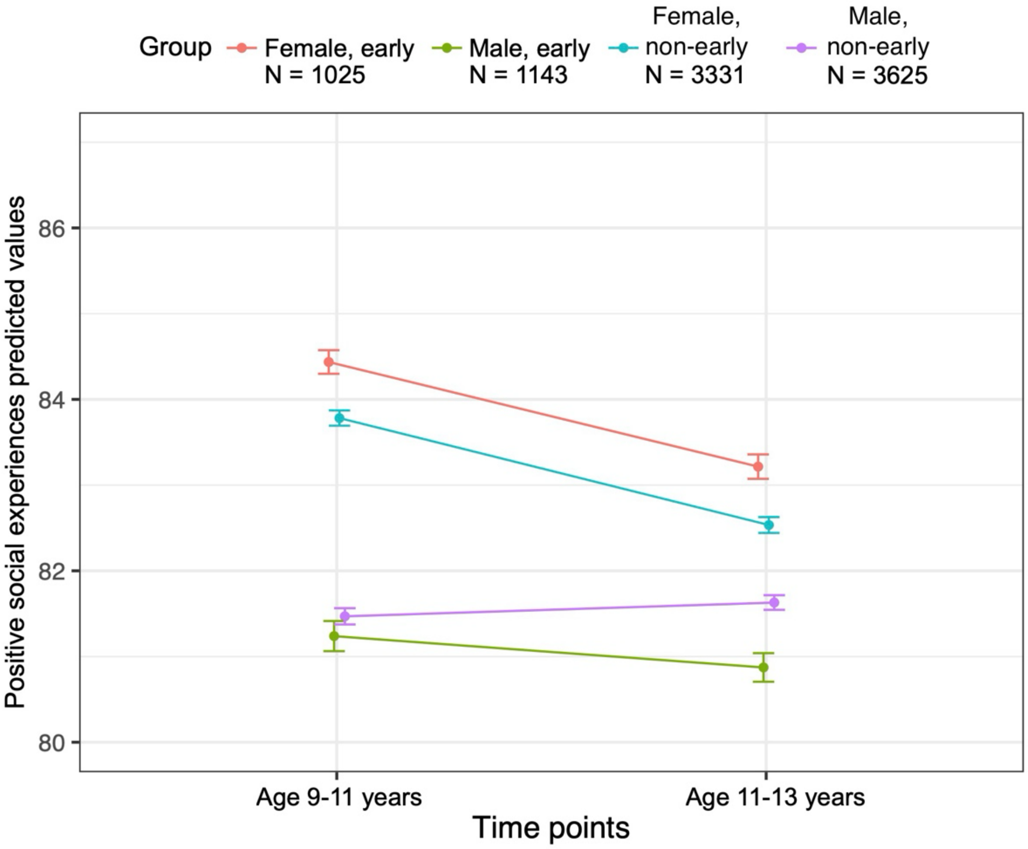

Supplement: Supplementary Material [file imag.a.27_supp.pdf]
